# Comparative genomics and transcriptomics analysis reveals evolution patterns of selection in the *Salix* phylogeny

**DOI:** 10.1186/s12864-019-5627-z

**Published:** 2019-03-29

**Authors:** You-jie Zhao, Xin-yi Liu, Ran Guo, Kun-rong Hu, Yong Cao, Fei Dai

**Affiliations:** 1 Key Laboratory of Forestry and Ecological Big Data State Forestry Administration, Southwest Forestry University, Kunming, 650224 Yunnan People’s Republic of China; 2 College of Big data and Intelligent Engineering, Southwest Forestry University, Kunming, 650224 Yunnan People’s Republic of China; 3 Key Laboratory for Forest Resources Conservation and Utilization in the Southwest Mountains of China, Ministry of Education, Southwest Forestry University, Kunming, 650224 Yunnan People’s Republic of China

**Keywords:** *Salix* phylogeny, Species migration, Comparative transcriptomics, Resistance gene, Selective evolution

## Abstract

**Background:**

Willows are widely distributed in the northern hemisphere and have good adaptability to different living environment. The increasing of genome and transcriptome data provides a chance for comparative analysis to study the evolution patterns with the different origin and geographical distributions in the *Salix* phylogeny.

**Results:**

Transcript sequences of 10 *Salicaceae* species were downloaded from public databases. All pairwise of orthologues were identified by comparative analysis in these species, from which we constructed a phylogenetic tree and estimated the rate of diverse. Divergence times were estimated in the 10 *Salicaceae* using comparative transcriptomic analysis. All of the fast-evolving positive selection sequences were identified, and some cold-, drought-, light-, universal-, and heat- resistance genes were discovered.

**Conclusions:**

The divergence time of subgenus *Vetrix* and *Salix* was about 17.6–16.0 Mya during the period of Middle Miocene Climate Transition (21–14 Mya). Subgenus *Vetrix* diverged to migratory and resident groups when the climate changed to the cool and dry trend by 14 Mya. Cold- and light- stress genes were involved in positive selection among the resident *Vetrix*, and which would help them to adapt the cooling stage. Universal- stress genes exhibited positive selection among the migratory group and subgenus *Salix*. These data are useful for comprehending the adaptive evolution and speciation in the *Salix* lineage.

**Electronic supplementary material:**

The online version of this article (10.1186/s12864-019-5627-z) contains supplementary material, which is available to authorized users.

## Background

Willows (genus *Salix*) are widely distributed in the northern hemisphere, ranging around the North Temperate Zone, and are the most important source of wood in forests [1–3]. *Salix* is a large and complex genus with about 450–520 species [1–4], which is under the spotlight with the genome projects’ completion of *Salix purpurea* [5] and *Salix suchowensis* [6]. Many studies have focused on this genus, particularly with regard to its phylogenetic relationships [7–15], the timing of diversification events [13, 15–18] and environmental stress tolerance [19–22]. Unfortunately, there is still a lot of controversy over the origin and speciation, divergence time and evolution patterns in the genus *Salix*.

A widely used classification system was proposed by *Skvortsov*, which divided the genus *Salix* into three subgenera *Salix, Vetrix* and *Chamaetia* [1]. The evidence of morphological taxonomy suggests that the subgenus *Vetrix* has passed two stages in its development [1]. When the climate became colder [23], the thermophilic groups either became extinct or moved south (Southern China and Southeast Asia), like Section *Eriostachyae, Daltonianae and Denticulatae* et al. Thus the hardy ones stayed and drastically expanded their ranges. At the same time, another younger and hardier formation of the subgenus *Salix* expanded across the northern hemisphere being represented by a number of boreal sections. However, no study explains how willows went through the long-distance migrations and how the resident and migratory groups adapted to the varied environments from high to low latitudes during the long evolutionary history.

Transcriptome sequencing technology can rapidly and economically obtain all RNA information of organisms at one time, which playing an important role in finding molecular markers and function genes for biology research [24, 25]. As more and more species had been completed transcriptome sequencing, comparative transcriptomics has received more attention from researchers [26–30]. Comparative transcriptomics can explain the phylogenetic relationships based on multiple species, and answer the functional differences between orthologous genes after species divergence in different living environment.

**Fig. 1:**
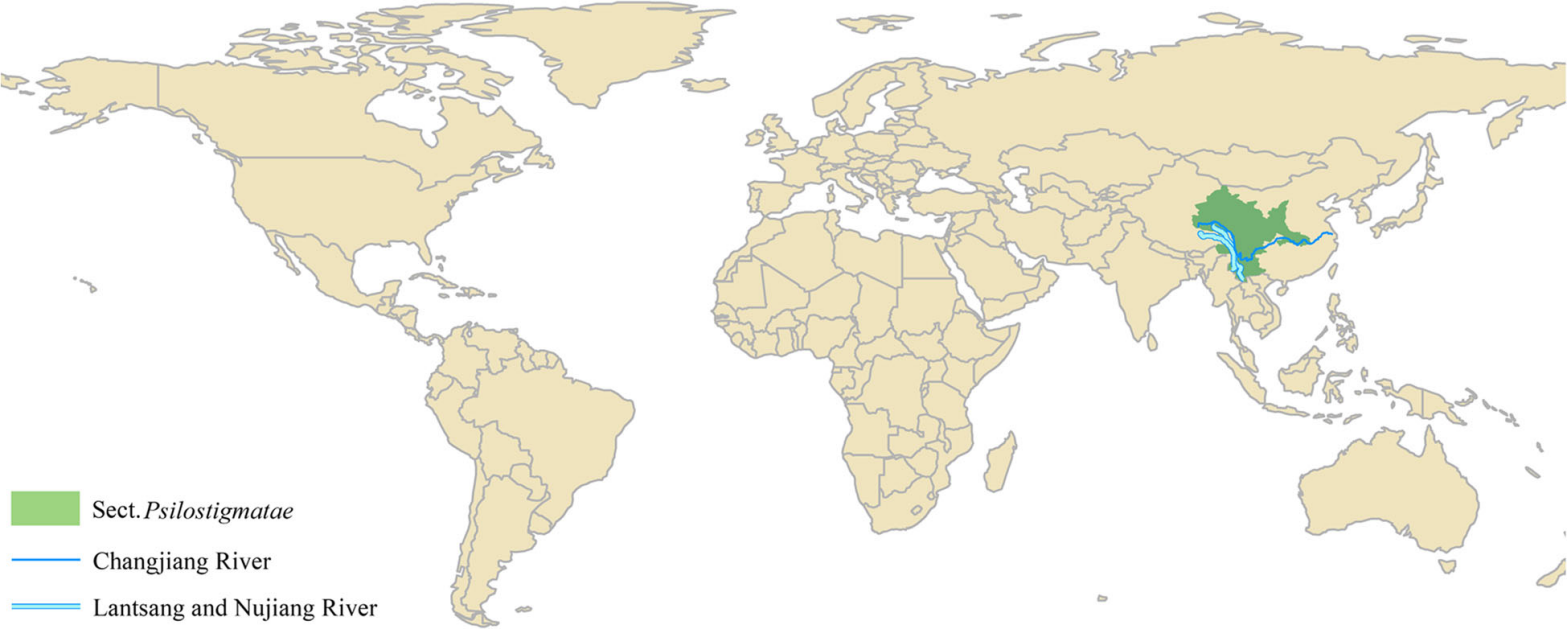
Geographical distribution of main species in section *Psilostigmatae*. The original information was from Flora of China (Additional file 1: Table S2)

In this study, transcript sequences of 9 *Salix* and one *Populus* [31] were downloaded from public databases (Table 1). Among them, *S. matsudana* and *S. babylonica* belong to subgenus *Salix,* other 7 willow species belong to subgenus *Vetrix*. Section *Psilostigmatae* (Fig. 1) named by Flora of China shares some species (like *S. salwinensis*) with migratory section *Daltonianae*, so *S. fargesii* of section *Psilostigmatae* is as the possible migratory species of *Vetrix*. Most species of *Vetrix* are mainly distributed in North China or further north areas except *S. fargesii* (Additional file 1: Table S1)*.* Comparative genomics and transcriptomics were subsequently analyzed in 10 *Salicaceae* species. A number of positive selection genes were found to be related to environmental factors in the *Salix* phylogeny.

**Table 1:** Data source of 9 *Salix* and one *populus* species. Main geographic distribution of 9 *Salix* is shown in Additional file 1: Table S1

*Salicaceae* species	Subgenus	Sect.	Data source	Sequencer
*S. purpurea*	*Vetrix*	*Helix*	JGI(v1.0)	Genome project
*S. suchowensis*	*Vetrix*	*Helix*	NJFU	Genome project
*S. sachalinensis*	*Vetrix*	*Vimen*	NCBI SRA (ERR2040399)	Illumina (Transcriptome)
*S. dasyclados*	*Vetrix*	*Vimen*	NCBI SRA (ERR2040396)	Illumina (Transcriptome)
*S. viminalis*	*Vetrix*	*Vimen*	NCBI SRA (ERR1558648)	Illumina (Transcriptome)
*S. eriocephala*	*Vetrix*	*Hastatae*	NCBI SRA (ERR2040397)	Illumina (Transcriptome)
*S. matsudana*	*Salix*	*Salix*	NCBI SRA (SRR1086819)	Illumina (Transcriptome)
*S. babylonica*	*Salix*	*Subalbae*	NCBI SRA (SRR1045959)	Roche 454 (Transcriptome)
*S. fargesii*	*Vetrix*	*Psilostigmatae*	NCBI SRA (ERR2040401)	Illumina (Transcriptome)
*P. trichocarpa*		*Tacmahaca*	JGI (v3.1)	Genome project

## Results

### Transcript sequences of 10 *Salicaceae* species

The average number of transcripts was about 40,649 in 10 *Salicaceae* species (Table 2), and *S. matsudana* had the largest number of unigenes (70,671), while *S. babylonica* had the least (3586). There are respectively 36,948, 26,599 and 37,865 annotated genes in the genomes of *P. trichocarpa, S. suchowensis* and *S. purpurea*. And these genes made up a total of 37 Mb, 34 Mb and 44 Mb cDNA sequences with a mean size of 1052 bp, 1344 bp and 1208 bp. More than 17,911 (28.2%), 8572 (32.2%) and 10,534 (27.8%) cDNAs has the length of > = 1500 bp in *P. trichocarpa, S. suchowensis* and *S. purpurea* (Additional file 2: Figure S1.)*.* By contrast, there are 47,753, 50,429, 36,191, 51,717, 70,617, 3586 and 45,719 unigenes in the transcriptomes of *S. sachalinensis, S. dasyclados, S. viminalis, S. eriocephala, S. matsudana, S. babylonica* and *S. fargesii,* which respectively made up a total of 29.1, 30.4, 30.2, 32.6, 54.0, 2.4 and 27.2 Mb sequences with a mean size of 638, 632, 874, 660, 802, 714 and 624 bp. And more than 77, 77, 61, 75, 62, 92 and 78% unigenes had the length of < 1000 bp in the transcriptomes of *S. sachalinensis, S. dasyclados, S. viminalis, S. eriocephala, S. matsudana, S. babylonica* and *S. fargesii*.

**Table 2:** Transcript sequences in 10 *Salicaceae* species

*Salicaceae* species	Number of sequences	Min length (bp)	Mean length (bp)	Max length (bp)	Total length (Mb)
*S. purpurea*	37,865	90	1208	16,419	43.66
*S. suchowensis*	26,599	150	1344	17,043	34.11
*S. sachalinensis*	47,753	300	638	5100	29.09
*S. dasyclados*	50,429	300	632	4143	30.44
*S. viminalis*	36,191	300	874	15,315	30.18
*S. eriocephala*	51,717	300	660	6303	32.57
*S. matsudana*	70,617	300	802	7065	54.04
*S. babylonica*	3586	300	714	3185	2.44
*S. fargesii*	45,719	300	624	4941	27.24
*P. trichocarpa*	36,948	84	1052	16,356	37.09

### SSRs identified in 10 *Salicaceae* species

A total of 3002, 2247, 1818, 1876, 1868, 2181, 3268, 234, 2139 and 2690 distinct SSRs were identified in *S. purpurea, S. suchowensis S. sachalinensis, S. dasyclados, S. viminalis, S. eriocephala, S. matsudana, S. babylonica, S. fargesii* and *P. trichocarpa,* and the incidences of different repeat types were determined (Table 3). Among the different classes of SSRs, the tri-nucleotide repeats were the most abundant (83, 81, 80, 81, 80, 80, 83, 48, 80 and 82%) and di-nucleotides were the second type (9, 11, 12, 12, 12, 13, 10, 36, 12 and 9%). Among the di-nucleotide repeats, AG/CT type showed the largest number in 10 *Salicaceae* species. Among the tri-nucleotide repeats, AGG/CCT type showed the largest number in *S. purpurea, S. suchowensis, S. sachalinensis, S. dasyclados, S. eriocephala* and *S. babylonica*, but AGC/CTG in *S. viminalis, S. matsudana* and *S. fargesii*.

**Table 3:** SSRs identified in 10 *Salicaceae* species

SSR type	*S. purpurea*	*S. suchowensis*	*S. sachalinensis*	*S. dasyclados*	*S. viminalis*	*S. eriocephala*	*S. matsudana*	*S. babylonica*	*S. fargesii*	*P. trichocarpa*
AC/GT	30	18	19	12	36	29	30	3	29	40
AG/CT	228	196	197	196	184	240	289	59	206	171
AT/AT	20	21	8	14	9	13	19	20	13	37
CG/CG	0	1	0	0	1	0	4	2	3	1
Di-nucleotide	278	236	224	222	230	282	342	84	251	249
AAC/GTT	75	58	92	74	64	119	75	2	80	122
AAG/CTT	403	323	221	274	238	275	430	23	252	377
AAT/ATT	45	21	17	19	28	33	43	8	32	44
ACC/GGT	506	377	319	267	255	327	469	21	353	409
ACG/CGT	88	77	65	90	61	88	164	9	60	69
ACT/AGT	17	10	4	7	11	8	9	1	7	16
AGC/CTG	495	360	224	236	318	291	605	13	362	405
AGG/CCT	545	378	328	330	294	384	463	21	345	390
ATC/ATG	208	150	153	162	154	197	292	9	141	296
CCG/CGG	113	70	46	47	64	44	116	6	89	76
Tri-nucleotide	2495	1824	1451	1515	1487	1739	2710	113	1721	2204
Tetra-nucleotide	13	6	3	8	11	8	10	12	5	12
Penta-nucleotide	11	10	8	9	7	13	10	3	14	15
Hexa-nucleotide	205	171	132	122	133	139	196	22	148	210
Total number	3002	2247	1818	1876	1868	2181	3268	234	2139	2690

### Orthologue identification and functional characterization between 10 *Salicaceae* species

**Fig. 2:**
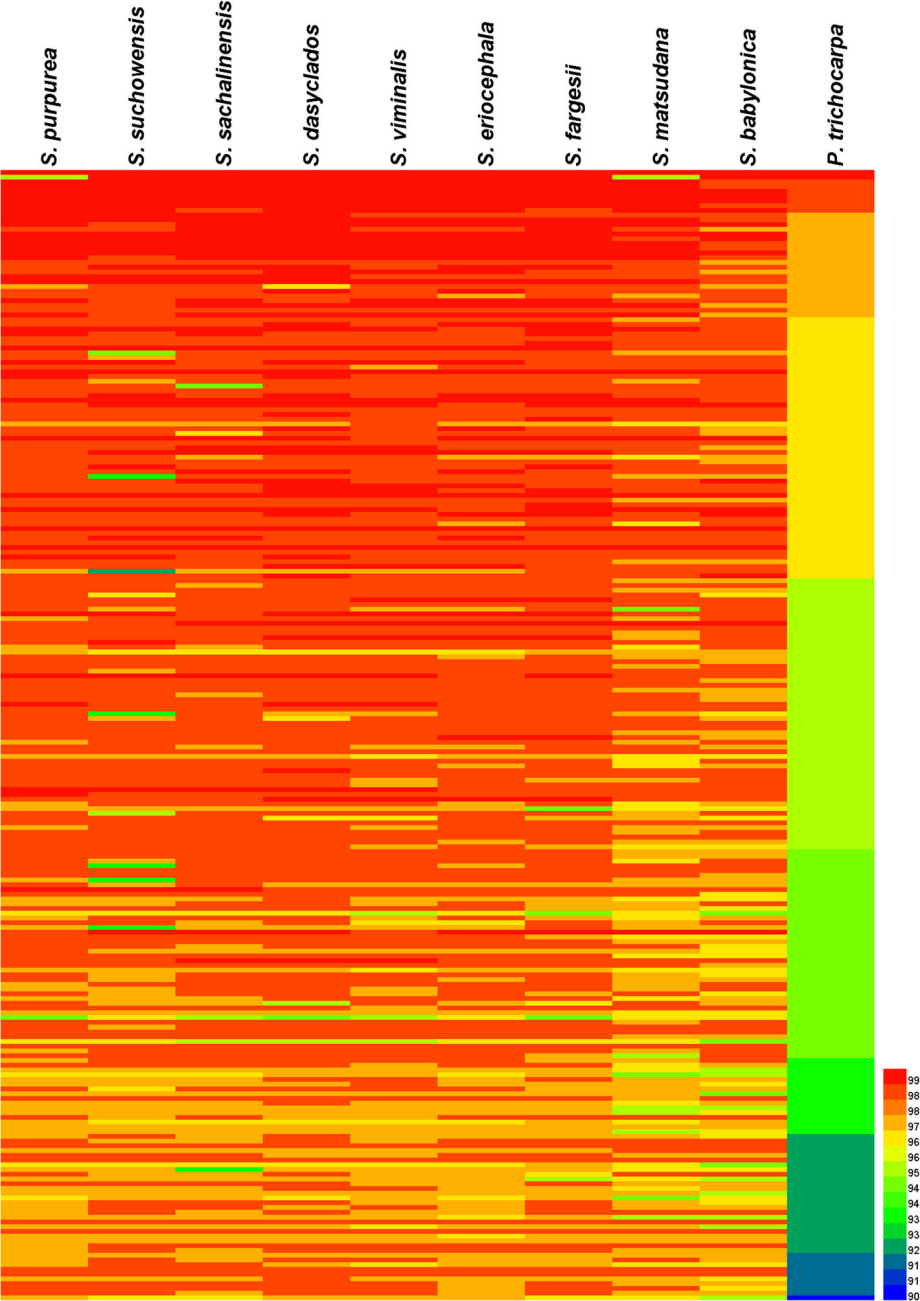
Functional annotation and divergence between orthologues of 9 *Salix* and one *Populus* species. The heat map is based on the 238 putatively orthologous transcripts of 10 species (Named orth1-orth238). The orthologues were annotated to different function with Gene Ontology (GO) (Additional file 1: Table S3). The sequences of 238 orthologues are shown in Additional file 3

All of the pairwise orthologues were identified by comparative analysis between the 10 *Salicaceae* species (Table 4). The results showed that *S. purpurea* had the maximum average number (6597) of orthologous genes, whereas *S. babylonica* had the minimum average number (707). The highest number of orthologous genes (9713) was found between *S. purpurea* and *S. suchowensis*, while the lowest number (681) was found between *S. babylonica* and *S. fargesii*. 238 single copy orthologues were found in all 10 *Salicaceae* species (Fig. 2). The orthologues were annotated with GO terms (Additional file 1: Table S3). Taking *P. trichocarpa* as an out-group species, the phylogenetic tree of *Salix* was constructed based on combined 238 orthologous transcripts using Maximum Likelihood (ML) method (Fig. 4).

**Table 4:** Number and Ks peaks of orthologous genes in 10 *Salicaceae* species

	*S. purpurea*	*S. suchowensis*	*S. sachalinensis*	*S. dasyclados*	*S. viminalis*	*S. eriocephala*	*S. fargesii*	*S. matsudana*	*S. babylonica*
*S. purpurea*									
*S. suchowensis*	9713/0.02								
*S. sachalinensis*	6319/0.02	5035/0.02							
*S. dasyclados*	6598/0.02	5221/0.02	5147/0.02						
*S. viminalis*	6898/0.03	5429/0.03	4812/0.03	4950/0.02					
*S. eriocephala*	6585/0.03	5238/0.03	5132/0.03	5301/0.02	4923/0.02				
*S. fargesii*	6258/0.03	4911/0.03	4669/0.02	4765/0.02	4638/0.02	4756/0.02			
*S. matsudana*	7934/0.04	6251/0.04	5217/0.04	5427/0.03	5322/0.04	5411/0.04	5078/0.04		
*S. babylonica*	892/0.05	696/0.05	708/0.04	714/0.04	688/0.03	713/0.04	681/0.03	726/0.02	
*P. trichocarpa*	8179/0.11	6246/0.12	4165/0.1	4325/0.11	4519/0.11	4400/0.11	4107/0.1	5317/0.11	549/0.11

### Phylogenetic analysis and divergence time

**Fig. 3:**
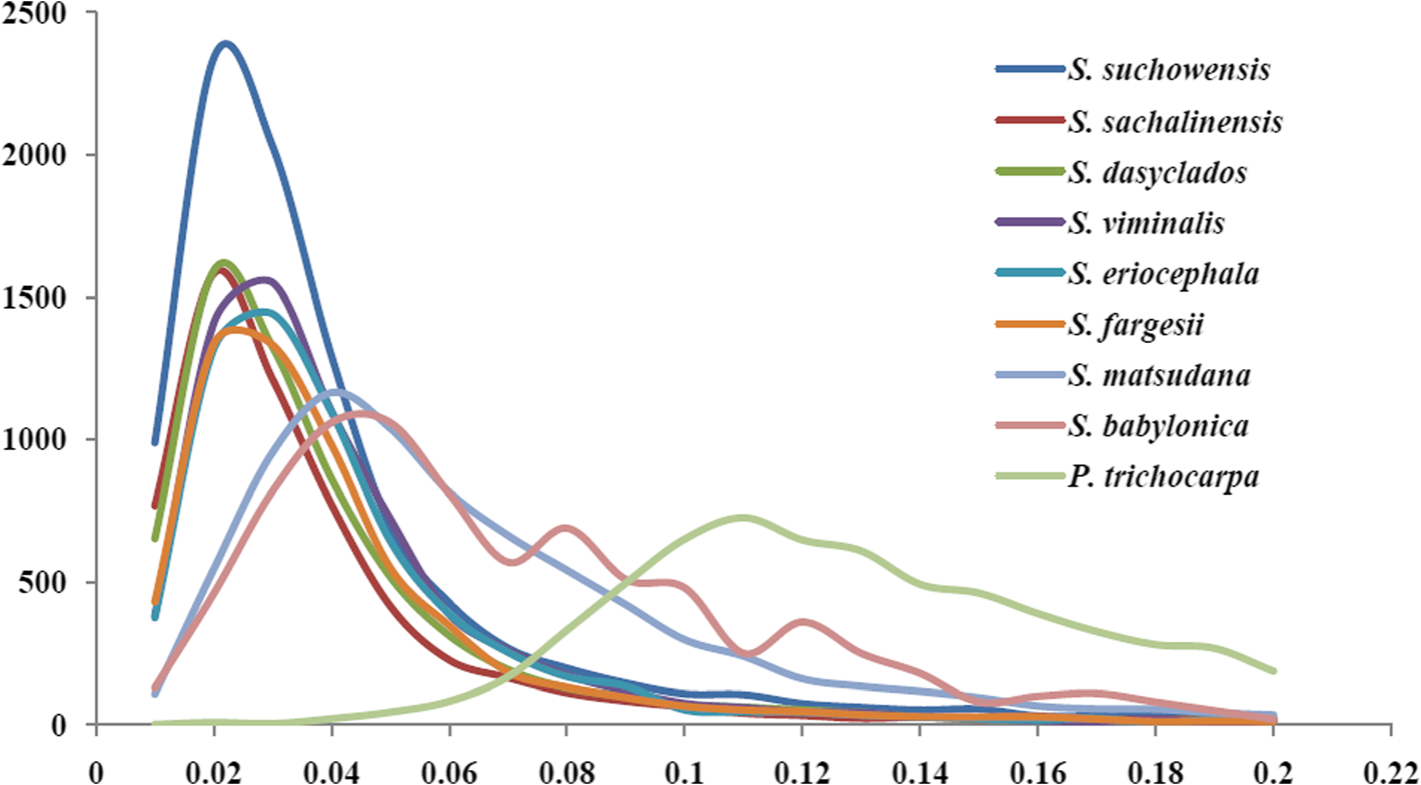
Distribution of Ks values of orthologous pairs between *S. purpurea* and others

The genetic distance of species was related to synonymous mutation rate calculated by orthologous genes, so the synonymous mutation rates of all pairs of orthologues were estimated in 10 *Salicaceae* species (Table 4). Between different branches (Fig. 3), *S. purpurea* has the Ks peak (0.02) with *S. suchowensis, S. sachalinensis* and *S. dasyclados*, 0.03 Ks peak with *S. viminalis, S. eriocephala* and *S. fargesii*, 0.04 Ks peak with *S. matsudana*, 0.05 Ks peak with *S. babylonica*, and the maximum Ks peak 0.11 with out-group *P. trichocarpa*. Between different genera, most of *Salix* species has the Ks peak 0.11*,* whereas *S. fargesii* was found the minimum Ks peak 0.10 with *P. trichocarpa* (Table 4). It is suggested *S. fargesii* was a relatively ancient species compared to others.

**Fig. 4:**
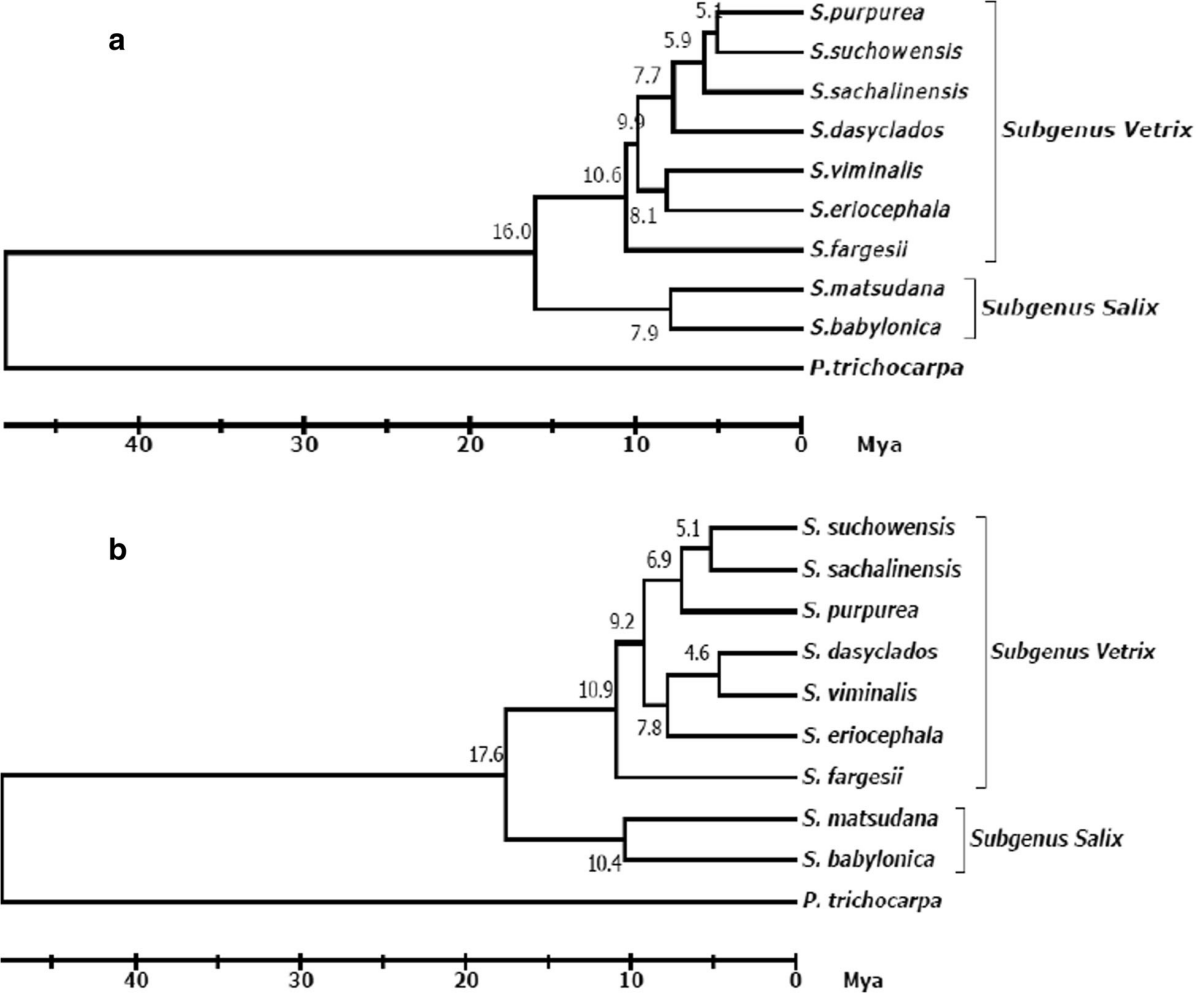
Phylogenetic tree of 9 *Salix* and one *Populus* species. **a** NJ tree. Phylogram derived using pairwise synonymous substitution rates of orthologous transcripts as a distance metric (not from multiple sequence alignments) and the neighbour-joining method. **b** ML tree. Phylogram derived using 238 single copy genes and the maximum-likelihood method. Original trees were shown in Additional file 4: Figure S2

Using *P. trichocarpa* as an out-group species, the phylogenetic tree of *Salix* was derived with the pairwise Ks values of the orthologous transcripts as a distance metric based on the neighbour-joining (NJ) method (Fig. 4). In the phylogenetic tree, the average Ks value is 0.11 between Genus *Salix* and Genus *Salix* (Calculated by Table 4), and which is nearly consistent with the value of 0.12 in previous studies [18]. Based on existing fossil evidence, the divergence time of genera *Salix* and *Populus* was about 48 million years old in middle Eocene sediments [16, 17]. With this time as the separation of the two lineages and K = 0.11, the rate of substitution (r) was calculated to about 1.14 * 10^− 9^ per site and year (T = K/2r), and which is very close to previous value of 1.28 * 10^− 9^ [18].

Using the fossil calibrations (48 Mya) of genera *Salix* and *Populus* [16, 17], the divergence times were estimated based on the 238 single copy genes and pairwise Ks distance metric of the orthologous transcripts (Table 4). The divergence of subgenus *Vetrix* and *Salix* occurred at about 17.6–16.0 Mya in the *Salix* phylogeny, and *S. fargesii* diverged at about 10.9–10.6 Mya with other species of subgenus *Vetrix* (Fig. 4). There were still some inconsistencies on the divergence time of subgenus *Vetrix* and *Salix* based on nuclear and plastome genes in previous studies [13, 15]. The time of 17.6–16.0 Mya between subgenus *Vetrix* and *Salix* supports the value of 16.9 Mya estimated by complete plastome genomes.

### Evolutionary pattern of *Salix* spp. genes

*Ka/Ks* rate of orthologous genes could reflect the evolution pattern of species. *Ka/Ks* > 1 indicates that the gene has involved in positive selection during evolution.

In the *Salix* phylogeny (Table 5), stress genes producing Glutathione S-transferase protein were generally found to be involved in positive selection between *S. purpurea* and *S. sachalinensis*, *S. purpurea* and *S. dasyclados, S. sachalinensis* and *S. dasyclados, S. viminalis* and *S. purpurea, S. viminalis* and *S. dasyclados, S. eriocephala* and *S. viminalis, S. eriocephala* and *S. dasyclados, S. matsudana* and *S. suchowensis, S. matsudana* and *S. fargesii.* Glutathione S-transferase protein could induce multiple stresses of cold-, drought-, salt- and oxidation- [32–34].

**Table 5:** Number and function annotation of positive selection genes in Genus *Salix*

	*S. purpurea*	*S. suchowensis*	*S. sachalinensis*	*S. dasyclados*	*S. viminalis*	*S. eriocephala*	*S. fargesii*	*S. matsudana*	*S. babylonica*
*S. purpurea*									
*S. suchowensis*	660								
*S. sachalinensis*	398/G	335							
*S. dasyclados*	454/CG	306/C	267/CG						
*S. viminalis*	414/G	270	254/L	270/G					
*S. eriocephala*	404	320	295	289/CG	281/G				
*S. fargesii*	414/CHU	302/H	260	281	255	242			
*S. matsudana*	351	242/G	210	238	207	212	257/UG		
*S. babylonica*	39	23	26	28	32	24	29	36/U	

C: Cold-stress; H: Heat-stress; L: Light-stress; U: Universal-stress; G: Multiple stresses by Glutathione S-transferase protein including Cold-, drought-, Salt- and Oxidation-; The Ka and Ks of resistance genes are shown in Additional file 1: Table S4. The sequences of resistance genes are shown in Additional file 5

In subgenus *Vetrix* except *S. fargesii* (Table 5), *S. dasyclados* was identified 454, 306, 267 and 289 positive selection genes with the spepies of subgenus *Vetrix*, *S. purpurea, S. suchowensis, S. sachalinensis* and *S. eriocephala.* Between them, cold- stress genes were found to be annotated to NP_190879.1, AAM23265.1, NP_849749.1 and AAN77157.1 (Additional file 1: Table S4)*,* which producing the proteins of P-loop NTPases [35], L-asparaginase [36], HOS10 with Myb damain [37] and thylakoid-bound ascorbate peroxidase [38]. 254 positive selection genes were identified between *S. sachalinensis* and *S. viminalis,* and one light-stress gene (NP_565524.1) was found by producing the SEP protein [39].

In subgenus *Salix* and *S. fargesii* (Table 5), 257 and 36 positive selection genes were identified between *S. matsudana* and *S. fargesii, S. matsudana* and *S. babylonica.* Universal-stress genes (NP_001132550.1 and NP_001132238.1) were wildly found between them by producing universal stress protein. Universal stress protein could induce by many environmental stressors such as nutrient starvation, drought, extreme temperatures, high salinity, and the presence of uncouplers, antibiotics and metals [40–42].

## Discussion

### *Salix* phylogeny derived by comparative genomics and transcriptomics

In previous studies, *Salix* phylogenies were usually derived by several nuclear and plastid markers [7–15]. Different markers always obtained different phylogenetic trees. Comparative genomics and transcriptomics could make use of more and more nuclear sequences. Phylograms were derived using two methods in this work. 238 single copy genes were strictly selected to construct the phylogenetic tree by maximum- likelihood method, which used most of the sites. Another method is based on the neighbor-joining method using the pairwise Ks values of the orthologous transcripts as a distance metric, which used most of the orthologous. The divergence times of subgenus *Vetrix* and *Salix* estimated by two methods were consistent with the value by complete plastome genomes [15]. It is improved that enough single copy nuclear sequences should obtain similar results with enough plastid sequences in the *Salix* phylogeny.

### Paleoclimate change in the divergence of *Salix* phylogeny

The divergence time of genera *Salix* and *Populus* was about 48 Mya at the period of Paleogene (66-23Mya) [43–45]. During the Paleogene, the global climate went against the hot and humid conditions of the late Mesozoic era and began a cooling and drying trend [23]. As the Earth cooled, tropical plants were restricted to equatorial regions and became less numerous. Deciduous plants became more common which could survive through the seasonal climates, during which *Salix* and *Populus* diverged.

Miocene (23 - 5Mya) is the main period in the divergence of *Salix* phylogeny (Fig. 4). During the period, there is evidence of a warm period from 21 Mya to 14 Mya named as the Middle Miocene Climate Transition (MMCT) [23], and the rare pleasant environment might cause the species diversity. The divergence time of subgenus *Vetrix* and *Salix* was about 17.6–16.0 Mya corresponding to the period of MMCT. Then global temperatures took a drop and some species were extinct by 14Mya [46–48], so the north subgenus *Vetrix* needed to migrate or adapt in order to survive. One group with *S. fargesii* diverged from subgenus *Vetrix* and migrated to south. The resident group of *Vetrix* had to adapt the cold and drought climate. By 8 Mya, the climate sharply cooled and formed the Quaternary Ice Age (2.6–0.1Mya) [49]. The climate change from MMCT to Quaternary Ice Age should play an important role in the divergence of *Salix* phylogeny.

### Universal- stress genes and migration of *S. fargesii*

The divergence time of *S. fargesii* (section *Psilostigmatae*) was about 10.9–10.6 Mya after the MMCT (21–14 Mya), and in which period the climate changed to be cooling. Wind and animal pollination had been proved to play an important role in the spread of willows [50–52]. Section *Psilostigmatae* are mainly distributed along the Changjiang (Yangtze) [53], Laantsang and Nujiang river of China (Fig. 1). The river provided the feasibility for the migration of the willow catkins by animal or other pollination, which is consistent with the distribution of Section *Psilostigmatae.* In previous studies, it was shown that the evolutionary history of the salix has involved multiple reticulation events that may mainly be due to hybridization [13]. Migration of *S. fargesii* maybe provided the possibility for the hybridization of *Salix*.

Our result shows that universal- stress genes were identified to be involved in positive selection between *S. fargesii* and subgenus *Salix*. It is suggested that selective evolution of universal- stress gene should play an import role in migrating to south for *S. fargesii.* When *S. fargesii* moved to south, universal- stress gene can help to produce the abiotic resistances to adapt the complex environment of south areas.

### Cold-, light- stress genes and the north resident group of sub genus *Vetrix*

After the MMCT (21–14 Mya), the global climate came back the cooling and drying trend [23]. In previous studies, it was shown that the evolutionary history of the *Salix* maybe affected by the profound climatic cooling during the Tertiary [13]. The resident group of section *Vetrix* had to adapt to the cooling stage especially the high-latitude. Cold-, light- stress genes were widely identified to be involved to positive evolution among *S. purpurea, S. suchowensis, S. sachalinensis*, *S. eriocephala* and *S. dasyclados.* It is suggested that the cool and dry climate had played an important role in the speciation of north group of Section *Vetrix.*

## Conclusions

In this study, we completed the comparative analysis based on genomic and transcriptomic sequences of 9 *Salix* and one populus species. All pairwise of orthologues were identified in these species, from which we constructed a phylogenetic tree and estimated the rate of diverse. The divergence times were estimated by the comparative analysis, and which suggested the speciation of *Salix* was involved in the period from MMCT (21–14 Mya) to Quaternary Ice Age (2.6–0.1Mya). The warm climate of MMCT might cause the divergence of subgenus *Vetrix* and *Salix*. Then global temperatures came back to the cool and dry trend by 14 Mya, so willows needed to migrate or adapt in order to survive. The phylogenetic relationship and geography distribution suggest that section *Psilostigmatae* might migrate from north to south by the Changjiang, Laantsang and Nujiang river of China. Universal- stress genes were involved in positive evolution and could help them to adapt to the south complex environment. Cold- and light- stress genes were identified to be involved in positive evolution among the resident *Vetrix*. It is suggested the resident *Vetrix* had to adapt to the cool and dry environment in order to survive. The study shows that the paleoclimate change and selective evolution had played an important role in the divergence of *Salix* phylogeny.

## Methods

### Data sources

In order to discover the evolutionary pattern of orthologues, the cDNAs and transcripts of 9 *Salix* and one *Populus* (out-group) were downloaded from the public databases (Table 1). The cDNAs of *P. trichocarpa* (v3.1), *Salix purpurea* (v1.0) and *S. suchowensis* were directly derived from the JGI [5] and willow genome project of NJFU (*http://bio.njfu.edu.cn/ss_wrky*). Transcriptome sequencing of *S. sachalinensis, S. dasyclados, S. viminalis, S. eriocephala, S. matsudana, S. babylonica* and *S. fargesii* were obtained from the SRA database of NCBI. Geographic distributions of section *Psilostigmatae* was draw by ArcGIS based on Flora of China (*http://frps.iplant.cn*) (Additional file 1: Table S2).

### Data filtering and de novo assembly

SRA datasets with FASTQ format were filtered to remove raw reads of low quality. Transcriptome assembly was achieved using the short-read assembly program Trinity [54]. The assembled sequences (> = 300 bp) were combined and clustered with CD-HIT (version 4.0) [55, 56]. Sequences with similarity > 95% were divided into one class, and the longest sequence of each class was treated as a unigene during later processing.

### Identification of SSRs in 10 *Salicaceae* species

Putative SSRs in Unigenes and cDNAs were identified by MISA software. The options of Di- to hexa-nucleotide SSRs were set to 6 (for di-), 5 (for tri- and tetra-), and 4 (for penta- and hexa-), and all SSRs were characterized in 10 *Salicaceae* species.

### Identification of orthologues among 10 *Salicaceae* species

OrthoMCL software [57] was used to cluster the transcribed sequences. Based on the proteins of *Salix purpurea* as reference, one-to-one sequences of each group were then filtered to use in subsequent analyses. The annotations obtained from Nr were processed through the BLAST2GO program [58] to get the relevant GO terms. Heatmap of orthologues was draw by R language.

### Estimation of synonymous substitution and non-synonymous substitution rates

In order to remove the unigenes without open reading frames, pair-wise orthologues were searched against plant protein sequences of GenBank with BLASTX tool. The method has been used in previous studies [59]. Clustalw software [60] was used to align the filtered pair-wise orthologues, and the output files were formatted to NUC format for subsequent analysis. The rates of synonymous substitutions (Ka) and non-synonymous substitutions (Ks) were estimated with PAML software [61].

### Phylogenetic analysis

There were still some inconsistencies on phylogenetic relationship in previous studies. Phylograms were derived using two methods in this work. Single copy genes by orthoMCL were aligned by Muscle [62] and formated by Gblock [63], maximum- likelihood method was used to build the phylogenetic tree by MEGA6 [64] (bootstrap is 1000 and Kimura 2-parameter model). Another method is based on the neighbor-joining method of MEGA6 [64] using the pairwise Ks values of the orthologous transcripts as a distance metric (Table 4). *Populus trichocarpa* was used as an out-group to root trees.

## Additional files

Additional file 1: **Table S1.** Main geographic distributions of 9 *Salix* used in this work. **Table S2.** Geographic distributions of main species in Sect. *Psilostigmatae*. **Table S3.** GO annotation of shared orthologues in 10 *Salicaceae* species. **Table S4.** Information of resistance genes involved in positive selection in Genus *Salix. (XLS 60 kb)*

Additional file 2: **Figure S1.** Length distribution of transcripts in 10 *Salicaceae* species. (TIF 2038 kb)

Additional file 3: Sequences of shared orthologues in 10 *Salicaceae* species. (FA 1803 kb)

Additional file 4: **Figure S2.** Original tree of NJ and ML methods. (TIF 1481 kb)

Additional file 5: Sequences of resistance genes in Genus *Salix*. (FA 26 kb)

## Abbreviations

COG: Clusters of orthologous groups; SSR: simple sequence repeat
GO: Gene Ontology
Ka: Non-synonymous substitutions per non-synonymous site
Ks: Synonymous substitutions per synonymous site
ML: Maximum- likelihood
MMCT: Middle Miocene Climate Transition
Mya: Million years ago
NJ: Neighbor-joining
