# Supplementary figures and images for: Comparative genomics and transcriptomics analysis reveals evolution patterns of selection in the Salix phylogeny
Source: BMC Genomics. 2019 Mar 29;20:253. doi: 10.1186/s12864-019-5627-z (PMC6440167; doi:10.1186/s12864-019-5627-z)

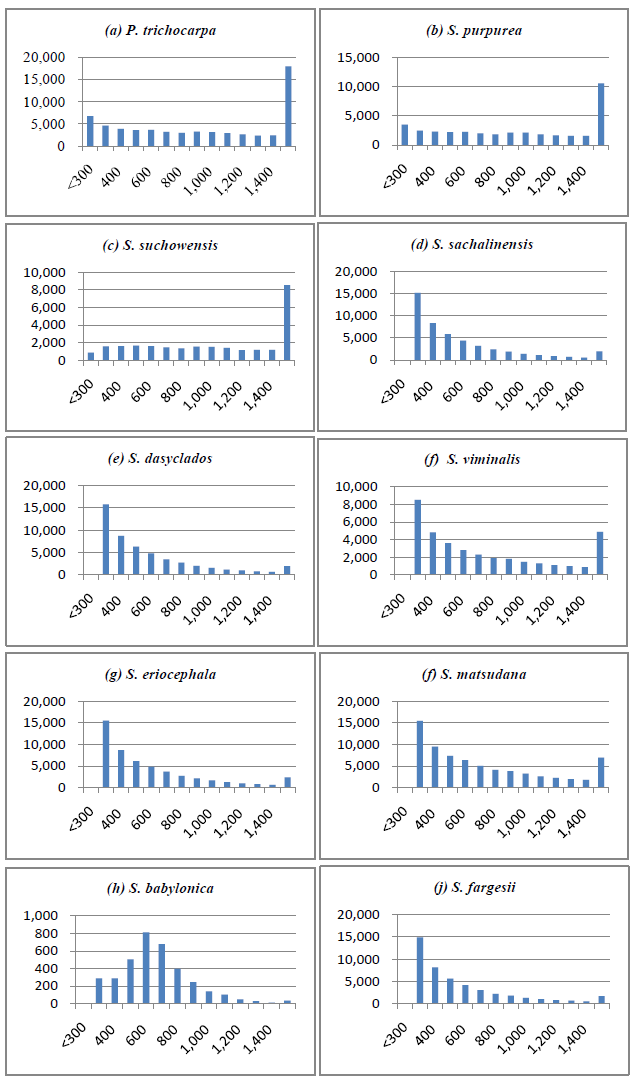

Supplement: Supplementary file 2 — Figure S1. Length distribution of transcripts in 10 Salicaceae species. (TIF 2038 kb) [file 12864_2019_5627_MOESM2_ESM.tif]

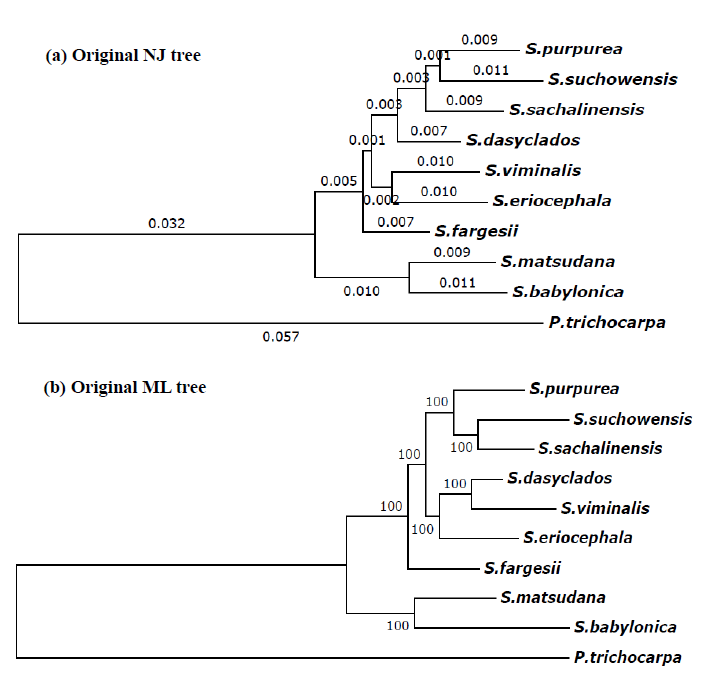

Supplement: Supplementary file 4 — Figure S2. Original tree of NJ and ML methods. (TIF 1481 kb) [file 12864_2019_5627_MOESM4_ESM.tif]
